# Effect of Semisolid Formulation of Persea Americana Mill (Avocado) Oil on Wound Healing in Rats

**DOI:** 10.1155/2013/472382

**Published:** 2013-03-19

**Authors:** Ana Paula de Oliveira, Eryvelton de Souza Franco, Rafaella Rodrigues Barreto, Daniele Pires Cordeiro, Rebeca Gonçalves de Melo, Camila Maria Ferreira de Aquino, Antonio Alfredo Rodrigues e Silva, Paloma Lys de Medeiros, Teresinha Gonçalves da Silva, Alexandre José da Silva Góes, Maria Bernadete de Sousa Maia

**Affiliations:** ^1^Department of Pathology, Federal University of Pernambuco, 50670-901 Recife, PE, Brazil; ^2^Department of Physiology and Pharmacology, Federal University of Pernambuco, 50670-901 Recife, PE, Brazil; ^3^Department of Histology and Embryology, Federal University of Pernambuco, 50670-901 Recife, PE, Brazil; ^4^Department of Antibiotics, Federal University of Pernambuco, 50670-901 Recife, PE, Brazil; ^5^Department of Physiology and Pharmacology, Laboratory of Pharmacology of Bioactive Products, Federal University of Pernambuco, 50670-901 Recife, PE, Brazil

## Abstract

The aim of this study was to evaluate the wound-healing activity of a semisolid formulation of avocado oil, SSFAO 50%, or avocado oil *in natura*, on incisional and excisional cutaneous wound models in Wistar rats. An additional objective was to quantify the fatty acids present in avocado oil. On the 14th day, a significant increase was observed in percentage wound contraction and reepithelialization in the groups treated with 50% SSFAO or avocado oil compared to the petroleum jelly control. Anti-inflammatory activity, increase in density of collagen, and tensile strength were observed inSSFAO 50% or avocado oil groups, when compared to control groups. The analysis of the components of avocado oil by gas chromatography detected the majority presence of oleic fatty acid (47.20%), followed by palmitic (23.66%), linoleic (13.46%) docosadienoic (8.88%), palmitoleic (3.58%), linolenic (1.60%), eicosenoic (1.29%), and myristic acids (0.33%). Our results show that avocado oil is a rich source of oleic acid and contains essential fatty acids. When used *in natura* or in pharmaceutical formulations for topical use, avocado oil can promote increased collagen synthesis and decreased numbers of inflammatory cells during the wound-healing process and may thus be considered a new option for treating skin wounds.

## 1. Introduction

Wound healing is a complex process involving different cell types, cytokines, growth factors, and the extracellular matrix with the purpose of swiftly reestablishing skin integrity [1–3]. This wound healing process occurs in three overlapping phases: inflammation, proliferation, and remodeling [4–11].

Hemostasis is followed after several hours by an inflammatory stage, during which cytokines and growth factors are secreted, and leucocytes and to a lesser extent other cell types are recruited to clean the wound. In the proliferative phase tissue repair occurs in response to the factors produced initially. Endothelial cells proliferate to form new blood vessels that are essential for supplying blood to the wound site. A proliferation of fibroblasts also occurs, thus establishing a proper wound bed for reepithelialization, which starts with the proliferation and centripetal migration of keratinocyte from the wound edges or from hair follicles and sweat glands in the remaining dermis. During the last phase, the following events occur: regression of capillaries, reorganization of the extracellular matrix, and restructuring of scar tissue, which may take many months if not years [12–14].

Previous studies have shown that the healing process may be modulated by fatty acids [8, 10]. Linolenic (18 : 3 *ω*-3), linoleic (18 : 2 *ω*-6), and oleic (18 : 1 *ω*-9) acids are precursors of eicosapentaenoic (EPA) (20 : 5 *ω*-3), arachidonic (AA) (20 : 4 *ω*-6), and eicosatrienoic acids (ETA) (20 : 3 *ω*-9) which are part of the structure of cell membrane phospholipids and serve as substrates for the synthesis of eicosanoids (inflammatory mediators), such as prostaglandins, thromboxanes, prostacyclins (via cyclooxygenase), and leukotrienes (via lipooxygenase) [15–20]. Eicosanoids formed from arachidonic acid, prostaglandin E2, thromboxane B2, and leukotriene B4 are proinflammatory inducers, more potent than those formed from EPA, prostaglandin E3, thromboxane B3, and leukotriene B5, which have anti-inflammatory effects [15, 18, 19, 21]. Considering that these families of fatty acids compete for the same enzyme, the proper balance between *ω*3, *ω*6, and *ω*9 is of great importance [18]. Depending on the *ω*3 : *ω*6 : *ω*9 ratio of the diet more proinflammatory or anti-inflammatory eicosanoids can be synthesized. Besides modulating the inflammatory response, eicosanoids also act in immunological responses, platelet aggregation, and cell growth and differentiation [22].

Avocado (*P. americana*) extract or oil *in natura* has been used in wound healing [23, 24], the treatment of psoriasis [25], wrinkles, and stretch marks [26, 27], as well as for their hepatoprotective actions [28]. The unsaponifiable fraction of this oil has regenerative properties of the epidermis [26, 27], besides improving scleroderma [29].

Avocado oil extracted from the pulp of the fruit is rich in polyunsaturated fatty acids (PUFAs), linoleic (6.1–22.9%) and linolenic acids (0.4–4.0%), and the monounsaturated fatty acid (MUFA), oleic acid (31.8–69.6%). It also contains *β*-sitosterol, *β*-carotene, lecithin, minerals, and vitamins A, C, D, and E [23, 30–34]. Therefore, the aim of this study was to evaluate the wound-healing activity of a semisolid formulation of avocado oil, SSFAO 50%, or avocado oil *in natura* on incisional and excisional cutaneous wound models in Wistar rats and to characterize the fatty acids present in avocado oil.

## 2. Materials and Methods

### 2.1. Extraction and Fatty Acid Characterization of the *In Natura* Avocado Oil

The *in natura* oil of avocado (fruit), Margarida variety, was extracted using hexane as extraction solvent, following the method described by Salgado et al. [33]. The phytochemical characterization of the oil *in natura* in terms of its composition of fatty acids was determined after converting them into methyl esters [35]. The sample was analyzed using a Thermo Trace Ultra GC (SHIMADZU, model GC-14B) apparatus equipped with a flame ionization detector and HP-20 (carbowax 20 m) capillary column (25 m × 0.32 mm × 0.3 *μ*m). The column temperature was initially set to 40°C for 1 min, then increased to 150°C at heating to 55°C/min, and finally increased to 220°C at 1.7°C/min. The injector and detector temperatures were 200 and 220°C, respectively. Nitrogen was used as the carrier gas at a flow rate of 1.0 mL/min; injection was in split mode (1 : 20), and the injection volume was 1.0 *μ*L of the test sample. A standard fatty acid methyl ester mixture (Supelco, USA) was used to identify fatty acid methyl esters by their retention time. Fatty acid data were expressed as percent of total peak area.

### 2.2. Pharmaceutical Formulation

 The semisolid formulation of avocado oil, SSFAO 50%, was composed of avocado oil and a vehicle (petroleum jelly), in sufficient amount to daily treatment, order of ensures formulation stability. This manipulation met the standards and quality control for medicines from the Synthesis of Substances of Therapeutic Interest Laboratory, Department of Antibiotics of the Federal University of Pernambuco. As a negative control, the formulation vehicle (petroleum jelly) was used, and as a positive control, an oil rich in essential fatty acids (EFA) (Curatec EFA). The pre-clinical toxicity tests conducted in our laboratory to evaluate the dermal and ocular irritation, sensitization, and toxicity (acute and subchronic) of an SSFAO or *in natura* avocado oil in rodents and lagomorphs did not show any clinical signs of toxicity.

### 2.3. Animals

A total of 64 adult Wistar rats, male and female, with ages between 3-4 months and weighing between 200–250 g, were used. The animals came from the vivarium of the Department of Physiology and Pharmacology of the Federal University of Pernambuco. The rats were kept individually in metabolic cages, in 12 h light/dark cycle and at a constant temperature (20 ± 2°C), with water and food *ad libitum*, throughout the experiment. The experiments were approved by the Ethics Committee for Animal Experimentation (number 23076.027831/2010-21) of the Federal University of Pernambuco, Recife, Brazil.

### 2.4. Excisional Wound Model and Treatments

 The excisional wound model (secondary intention) is adequate for biochemical and histological assessment of healing [36]. The cutaneous wound model was performed as described previously [37, 38]. Twenty-four Wistar rats, male and female, were intramuscularly anesthetized with xylazine (3 mg kg^−1^) and ketamine (10 mg kg^−1^ i.m.) [39], followed by manual trichotomy and antisepsis with 0.1% iodine alcohol at the dorsal midline of the cervical region. A circular area of approximately 78.5 mm^2^ of skin (subcutaneous tissue and fascia) was surgically removed using straight iris scissors and Adson forceps. A containment ring made of nontoxic and hypoallergenic silicone was sutured into place, with six stitches arranged symmetrically using 4.0-nylon monofilament, so that the surgical wound would remain in the center [38].

Injured rats were randomly divided into four groups (*n* = 6), with each group receiving a different treatment. The wound was treated with topical application (±100 mg) of 50% SSFAO, *in natura* avocado oil, EFA (positive control), or petroleum jelly (negative control), once daily for 14 consecutive days.

### 2.5. Macroscopic Analysis

Area of wounds at each time point was determined by the formula: *A* = *π* · *R* · *r*, where “*A*” represents the area (mm^2^), “*R*” larger radius, and “*r*” smaller radius [38]. Thus, percentage wound contraction at each time point was derived by the following formula: percent wound contraction = (initial wound area − current area)/initial wound area × 100% [40]. Measurements were made daily by the same examiner using a digital caliper, with the animals under physical restraint. The crusts of the wounds were removed on the seventh day to allow the evaluation of the accurate value of the remaining wound area and the tissue that was below them. The presence of granulation tissue, exudate and fibrin (slough), and crust formation as well as reepithelialization were also evaluated. 

### 2.6. Histopathological and Histomorphometric Analysis

At 15 days after operation, animals were euthanized in a CO_2_ chamber to allow the collection of treated sites for the histopathological and histomorphometric analysis. The samples was fixed in 10% buffered formalin for 24 hours, and subsequently dehydrated in ethanol, cleared in xylene, embedded in paraffin wax, and sliced with a cryotome (Minot-type). Tissue sections (thickness 4 **μ**m) were placed on slides previously coated with Mayer's albumin and after drying were stained with hematoxylin and eosin for morphological assessment [41, 42] and with Masson's Trichrome to collagen analysis [42, 43]. The slides were examined with a light microscope combined with a digital camera (Olympus BX-49); five images per field (0.0018 mm^2^) were captured (total magnification 400x) to obtain the mean. The images (pixel resolution 480 × 752) were stored and subjected to counting of inflammatory cells, fibroblast cells, number of blood vessels, and collagen density with the aid of ImageJ software (National Institutes of Health, USA). In the RGB system (red-green-blue), the values for blue were used for determinate collagen density, where the blue area was mathematically divided by the RGB area and multiplied by 100%. 

### 2.7. Incisional Wound Model and Treatments

The incisional wound model (first intention) is excellent for biomechanical analysis of wound strength [36]. Forty Wistar rats, male and female, were intramuscularly anesthetized with xylazine (3 mg kg^−1^) and ketamine (10 mg kg^−1^ i.m.) [39], followed by manual trichotomy and antisepsis with 0.1% iodine alcohol in the region of the dorsal midline. Afterwards, incisional wounds 3 cm in length were made in the skin using a number 15 scalpel. The site was dissected with blunt dissection to offset the adjacent muscle-aponeurotic plane, then repositioned, and sutured with two simple stitches using 4.0-nylon monofilament. After the operation, the rats were randomly divided into four groups (*n* = 10) and treated with topical application (±100 mg) of 50% SSFAO, *in natura* avocado oil, EFA (control positive), or petroleum jelly (negative control), once daily for 10 consecutive days. The sutures were removed on the eighth day. On the 11th day after operation, the animals were euthanized in a CO_2_ chamber and a 3 × 6 cm fragment of skin around the wound site was removed. The tensile strength of the fragments was analyzed by an EMIC tensiometer, model DL-500 MF, maximum capacity 5 kN. Forces were applied, perpendicular to the scar at a speed of 5 mm/min. The maximum strength was considered to be the greatest force or charge applied to the fragment until it ruptured.

### 2.8. Statistical Analysis

Results were expressed as means ± SEM and subjected to one-way ANOVA (inflammatory cells, fibroblast cells, number of blood vessels, collagen density, percent contraction wound, and tensile strength of the skin) or two-way ANOVA (presence of crust, exudate, fibrin-slough, and tissue reepithelialization) with Bonferroni's posttest comparing the treatment groups with SSFAO 50% or *in natura* avocado oil to the EFA and petroleum jelly controls, considering values of *P* < 0.05 statistically significant. All statistical analyses were performed using Graph Pad Prism Instant, version 5.0 (GraphPad Software, San Diego, CA/USA).

## 3. Results

### 3.1. Fatty Acid Characterization in the Avocado Oil *In Natura*

The fatty acid (FA) methyl esters were identified using GC, and the relative content of each component was determined by the peak area normalization method. The following ratio was observed: 24.00% saturated fatty acid (SFA), 52.06% monounsaturated fatty acid (MUFA), and 23.94% polyunsaturated fatty acid (PUFA). This study indicated that avocado oil has a high content (47.20%) of oleic acid (C18 : 1n9c), followed by (23.66%) palmitic acid (C16 : 0), (13.46%) linoleic acid (C18 : 2n6c), (8.88%) cis-13, 16-docosadienoic acid (C22 : 2), (3.58%) palmitoleic acid (C16 : 1), (1.60%) linolenic acid (C18 : 3n3), (1.29%) cis-11-eicosenoic acid (C20 : 1), and (0.33%) myristic acid (C14 : 0).

### 3.2. Development of Excisional Wounds

**Figure 1:**
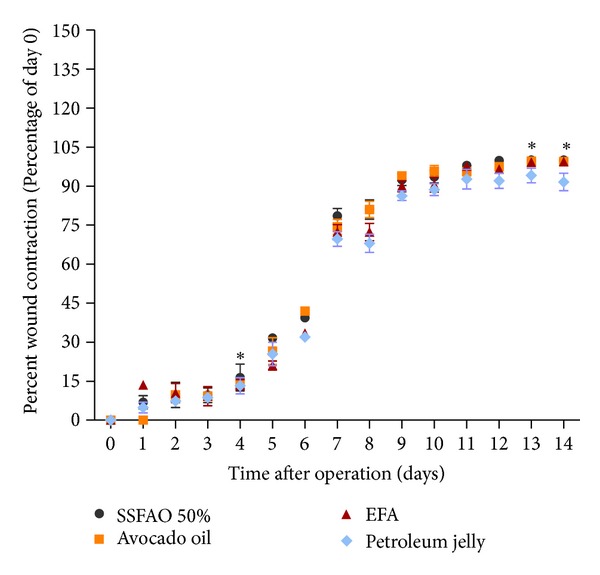
Percentage of wound contraction during the experimental period of treatment groups with SSFAO 50%, *in natura* avocado oil, EFA (control positive), or petroleum jelly (control negative). On the fifth day, SSFAO 50% or avocado oil groups maintained the percentage of contraction of wound slower, when compared to the EFA control. However, on the 14th day, there was a significant improvement in percentage wound contraction in the SSFAO 50% or *in natura* avocado oil groups when compared to the petroleum jelly control. Data are shown as average ± SEM (*n* = 06). *P < 0.05 versus controls, **P < 0.01 versus controls, and ***P < 0.001 versus controls.

To evaluate whether the treatment with SSFAO 50% or *in natura* avocado oil influenced the time for excisional wound closure, daily measurements were taken of all animals. On the fifth day of treatment, a significant difference (*P* < 0.05) was observed in percentage wound contraction in groups treated with 50% SSFAO (6.92 ± 2.55%) and *in natura* avocado oil (0.00 ± 0.00%) when compared to the EFA control (13.52 ± 1.25%). However, on the 13th day, there was a significant improvement in percentage wound contraction in the 50% SSFAO treated group (100.00 ± 0.00%) when compared to the petroleum jelly control (94.14 ± 2.81%). On the 14th day there was a significant increase percentage wound contraction in the groups 50% SSFAO (100.00 ± 0.00%) and *in natura* avocado oil (99.52 ± 0.48%) when compared to petroleum jelly control (91.63 ± 3.35%, % wound contraction of day 0) (Figure 1). A significant increase (*P* < 0.05) in the presence of crust was observed on the second day treatment, in the group treated with SSFAO 50% or *in natura* avocado oil (six animals—100%) compared to the EFA control group (0%).

After removal of the crust (seventh day), a significant decrease (*P* < 0.05) was observed in the presence of fibrin (slough) in the groups treated with SSFAO 50% or *in natura* avocado oil (0%) compared to the petroleum jelly control (two animals—33.33%). The presence of serous exudate was observed in the early postoperation hours in all animals. A significant decrease (*P* < 0.05) in the presence of exudate was seen in the seventh day in the group treated with *in natura* avocado oil (four animals—66.66%) compared to the petroleum jelly control (six animals—100%). At the other time periods, there was no significant difference these variables when comparing the treatment groups to the controls.

### 3.3. Histomorphometric Analysis of Excisional Wounds

**Figure 2:**
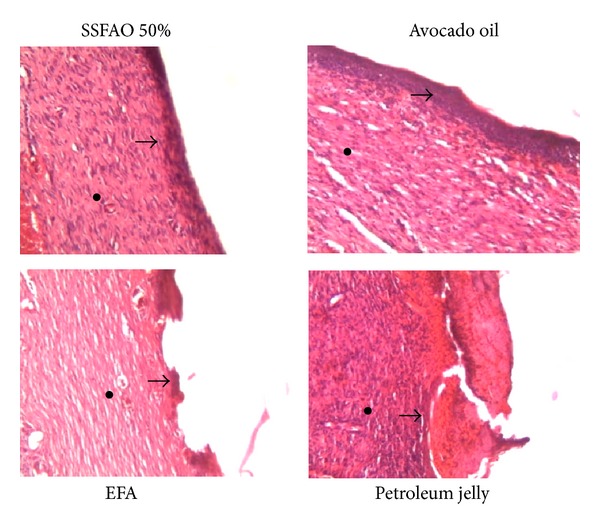
Histopathologic observation of treated excisional wound with SSFAO 50%, *in natura *avocado oil, EFA (control positive) or petroleum jelly (control negative) at the 14th day after operation. Skin sections show the hematoxylin and eosin stained epidermal (asterisk) and dermal (arrow) (40x magnification). Photographs are showing clear evidence for epithelization, keratinização and scar area formation in treated groups with SSFAO 50% or avocado oil. Data are shown as average ± SEM (*n* = 06). *P < 0.05 versus controls, **P < 0.01 versus controls, and ***P < 0.001 versus controls.

To determine the effect of SSFAO 50% or *in natura *avocado oil on tissue repair of excisional wounds, histopathological and histomorphometric analyses were performed on all animals (number of fibroblast and inflammatory cells, number of blood vessels, collagen density, and reepithelialization) of the dermal region treated for 14 days. A significant increase was observed in reepithelialization in the groups treated with 50% SSFAO or *in natura* avocado oil (six animals—100%) compared to the petroleum jelly control (three animals—50%) (Figure 2).

**Figure 3:**
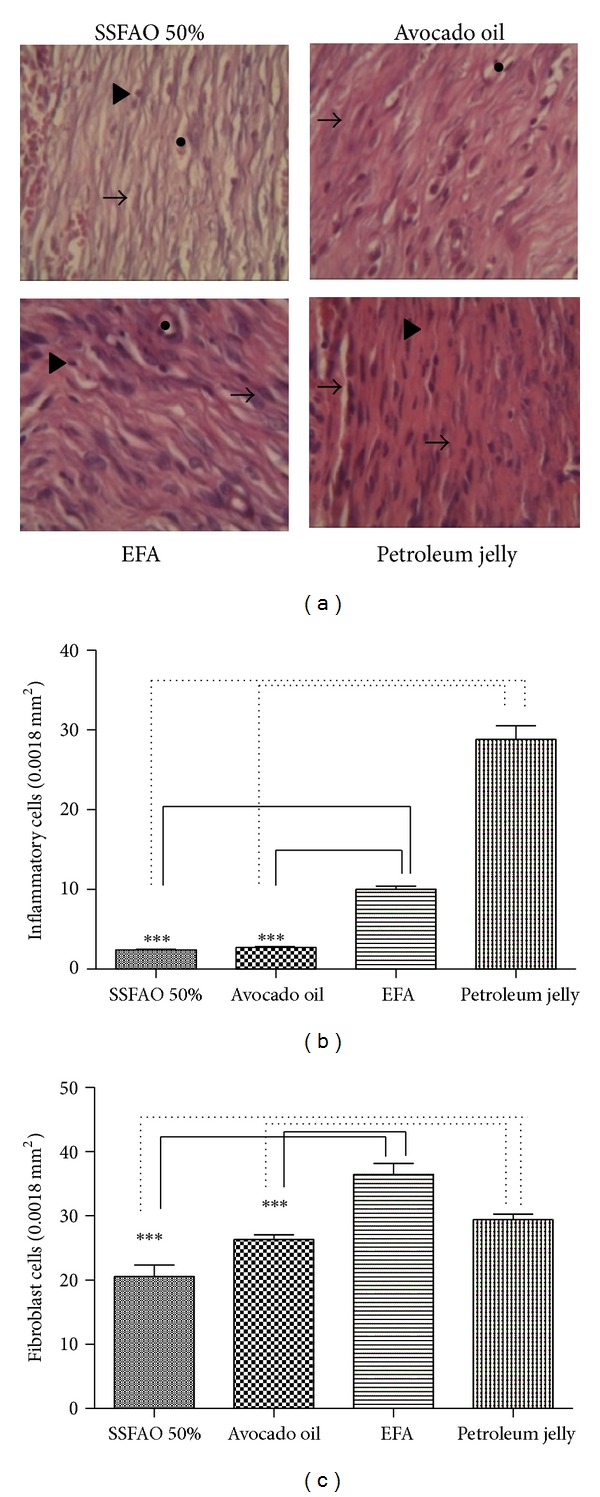
Histopathologic observation of treated excisinal wound with SSFAO 50%, *in natura *avocado oil, EFA (control positive), or petroleum jelly (control negative) at the 14th day after operation. Tissue sections were stained with hematoxylin and eosin (400x magnification). (a) Representative images show granulation tissue presenting fibroblasts (arrow) and inflammatory cells (arrowheads) and surrounding capillaries (asterisk). Fewer inflammatory (b) and fibroblast (c) cells are seen in treated excisinal wound with SSFAO 50% or *in natura *avocado oil when compared to controls. At least 30 different random fields were measured per treatment (*n* = 6). Data are shown as average ± SEM. *P < 0.05 versus controls; **P < 0.01.

Histopathological and histomorphometric analyses showed anti-inflammatory action in groups treated with SSFAO 50% or *in natura* avocado oil (2.50 ± 0.15 cells, 2.71 ± 0.12 cells, resp.) when compared with the EFA control (10.00 ± 0.41 cells) or petroleum jelly control (28.82 ± 1.70 cells, resp. (Figures 3(a) and 3(b)). A significant decrease (*P* < 0.05) was observed in the number of fibroblast cells in the group treated with 50% SSFAO (20.56 ± 1.80 cells) when compared with the EFA control (36.45 ± 1.68 cells) or petroleum jelly control (29.37 ± 0.88 cells), respectively. Also observed a significant decrease in the fibroblast cells in the group treated with *in natura* avocado oil (26.30 ± 0.73 cells) when compared with EFA control (36.45 ± 1.68 cells) (Figures 3(a) and 3(c)).

**Figure 4:**
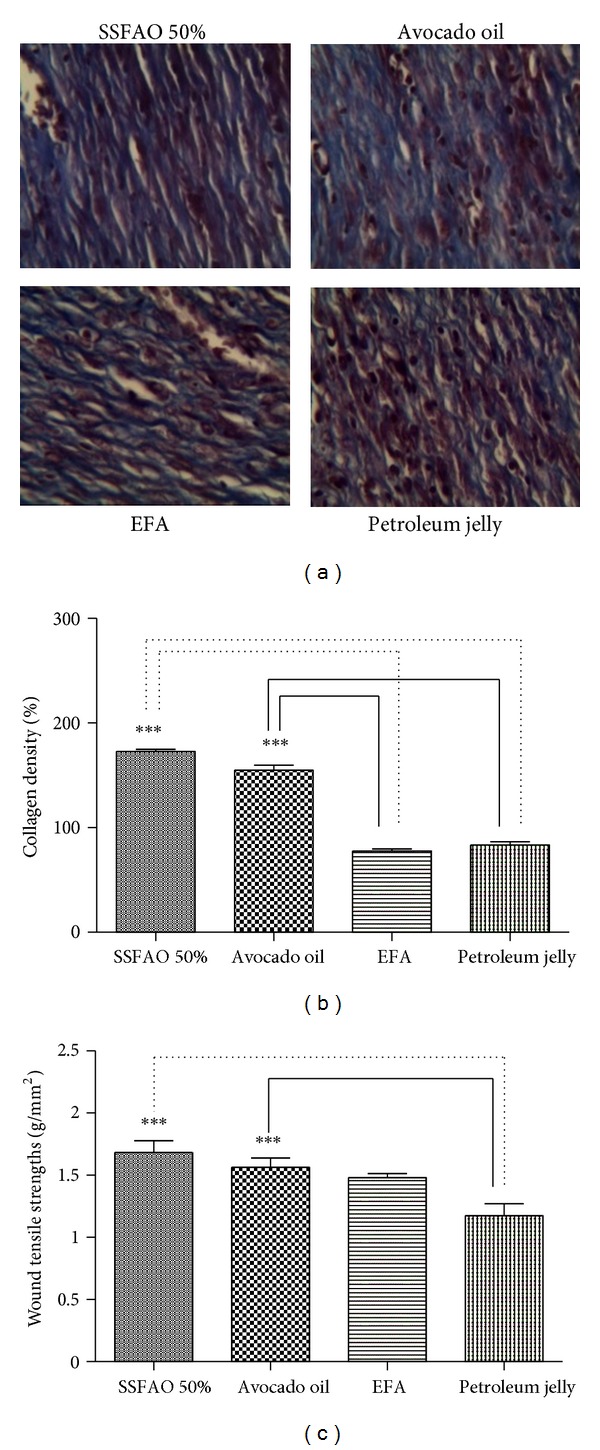
Histopathologic observation of treated wound with SSFAO 50%, *in natura *avocado oil, EFA (control positive), or petroleum jelly (control negative). (a) Tissue sections stained with Masson's Trichrome (400x magnification) to collagen fibers. Greater collagen deposition (b) and tensile strength (c) are seen in treated wound with SSFAO 50% or *in natura *avocado oil when compared to petroleum jelly control. Data are shown as average ± SEM. *P < 0.05 versus controls, **P < 0.01 versus controls, and ***P < 0.001 versus controls.

The group treated with 50% SSFAO or *in natura* avocado oil showed a significant increase (*P* < 0.05) in collagen density (41.89 ± 1.94% or 38.92 ± 1.12% resp.), when compared with the EFA control (31.04 ± 0.25%) or petroleum jelly control (34.08 ± 0.18%), respectively (Figures 4(a) and 4(b)). No difference was observed in the number of blood vessels among SSFAO 50% or avocado oil groups when compared to controls.

### 3.4. Tensile Strength Analysis of Incisional Wounds

On the tenth day after operation, the tensile strength of the scar tissue was measured using EMIC tensiometer. A significant increase (*P* < 0.05) was observed in tensile strength in the SSFAO 50% or *in natura *avocado oil groups (1.68 ± 0.09 g/mm^2^ or 1.56 ± 0.07 g/mm^2^, resp.), when compared with the petroleum jelly control (1.17 ± 0.10 g/mm^2^) (Figure 4(c)).

## 4. Discussion

Extracts of avocado (*P. americana* Mill.) have been used in wound healing [23, 24]. We note that the *in natura* avocado oil is rich in monounsaturated fatty acids, with oleic acid being the most prevalent, which corroborates with the results obtained by Tango et al. [31]. The linoleic and oleic acid contents are just shy of those described by Salgado et al. [33]; however, the amount of linolenic acids are above those verified by these authors. This fact can be caused by the anatomical region of the fruit, maturation stage, and geographic location of the growth of the plant [44, 45]. 

Fatty acids (oleic, linoleic, and linolenic) have been the subject of several studies, because they seem to be active in the healing process [10, 38]. The healing process can be monitored by assessing the rate of contraction of the wound, period of reepithelialization, tensile strength, and histopathology in different wound models [46]. We note that the rate of contraction of excisional wounds of animals treated with SSFAO 50% or avocado oil (fifth day) was slower than that present in the EFA control. A result similar to that was described by Franco et al. [38], who reported a significant delay in the contraction of the wounds, in the inflammatory stage of healing, in experimental groups compared to the EFA control. Probably, the delay in the contraction rate is related to the easy absorption of avocado oil through the skin [26, 27] allowing the wound bed to remain more exposed to the environment, increasing the chances of dehydration [38].

The best profile in the rate of contraction of wounds of animals treated with 50% SSFAO or avocado oil (13th and 14th days) is probably related to the properties of the avocado oil (PUFA, MUFA, *β*-sitosterol, *β*-carotene, lecithin, minerals, and vitamins A, C, D, and E), which encouraged the migration, proliferation, and cell differentiation during the proliferative phase of wound healing. This finding corroborates those of Nayak et al. [23] and Vega et al. [24], which demonstrated the effectiveness of topical or oral administration of an extract from avocado fruit in different types of wounds using rats.

In this study, the presence of devitalized tissue (slough) was not verified in animals treated with SSFAO 50% or avocado oil, unlike animals treated with petroleum jelly. Hess and Kirsner [47] attributed the presence of devitalized tissue in the wound bed to tissue changes caused by oxygen, drying of the wound bed, or high microbial density. We suggest that the lack of development of slough in the groups treated with 50% SSFAO or avocado oil is associated with the antimicrobial activity attributed to linoleic acid [38, 48], as well as the proper maintenance of hydration and oxygen stress in the wound bed.

The histopathological assessment revealed that the animals treated with 50% SSFAO or avocado oil *in natura* showed a significant increase in the presence of epithelial tissue. The possible pharmacological effects attributed to avocado oil, in regard to the healing process, can be associated with its phytochemical compounds, such as vitamins (A and E) and fatty acids (oleic, linoleic, and linolenic acids). As these fatty acids are precursors of pharmacologically active substances, such as prostaglandins, thromboxanes, prostacyclins, and leukotrienes [15–20] that are involved in regulating cell division and differentiation, angiogenesis and synthesis of the extracellular matrix [22, 40, 49]. As does linoleic acid [50], vitamin E has important antioxidant functions [51] in combating free radicals that are responsible for the cytotoxicity and delay in tissue healing [52]. The adequate availability of these products provides a favorable environment to reepithelialization when administered to the wound bed.

Topical application of 50% SSFAO or avocado oil *in natura* promoted a reduction in the number of inflammatory cells in the scar tissue, characterizing anti-inflammatory activity. The modulation of the inflammatory response can be attributed to the high availability of oleic acid present in the SSFAO, since this fatty acid induces a less intense local inflammatory response, and competes with linoleic and linolenic acids for the same enzymes (cyclooxigenases and lipooxigenases) synthesizing less powerful inflammatory mediators than those formed by arachidonic acid [15, 18, 19, 21].

A significant decrease was observed in the number of fibroblast cells in animals treated with 50% SSFAO or *in natura* avocado oil; however, the collagen deposition was inversely proportional, characterizing the maturing of scar tissue (remodeling phase). 

There is a view that, in the physiological process of healing, collagen accumulates in the area of the wound until the 21st day after the injury; after this period, the balance between synthesis and degradation of collagen is restored [53], with a rapid disappearance (apoptosis) of fibroblastic cells [54].

A significant increase was observed in the tensile strength; this was proportional; the deposition of collagen, in animals treated with 50% SSFAO or *in natura* avocado oil. This finding is backed by Nunes et al. [55], Stoff et al. [56], Deodhar [57], and Udupa et al. [58], who reported that the resistance of the skin is related to formation, concentration, and chemical reorganization of the collagen fibers during the remodeling stage. According to Hunt [59], Stoff et al. [56], and López et al. [60], the tensile strength test is used to describe the quality of the healing from incisional wounds, this being one of the most reliable ways. Thus, the increase in tensile strength observed in this study may be due to increased collagen synthesis or due to a change in the maturation process, result of the action of mono- and polyunsaturated fatty acids present in avocado oil.
